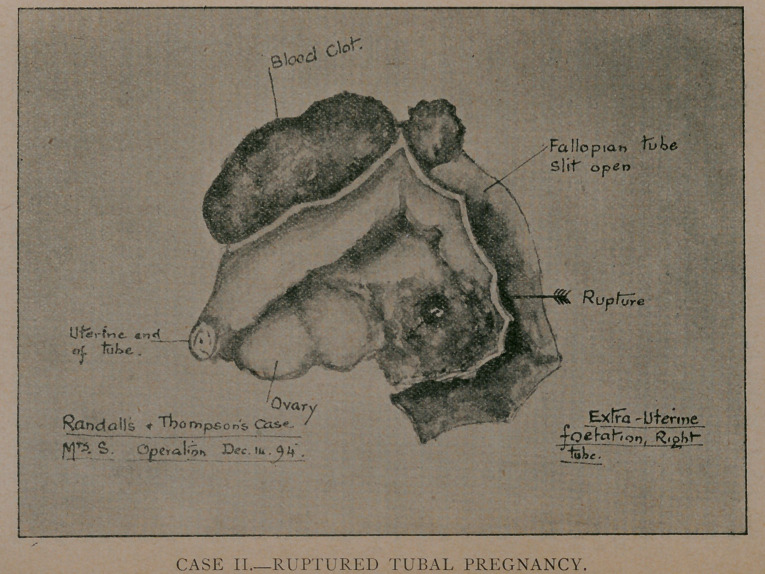# Notes of Two Cases of Extra-Uterine Pregnancy

**Published:** 1895-12

**Authors:** James E. Thompson

**Affiliations:** M.B., B.S., LOND.; F.R.C.S., ENG. Professor of Surgery, University of Texas


// TEXAS MEDICAL JOURNAL.

ESTABLISHED JULY, 1885.

PUBLISHED MONTHLY.—SUBSCRIPTION $2.00 A YEAR.

VOL. XI. AUSTIN, DECEMBER, 1895. NO. 6.

### Original Contributions.

For Texas Medical Journal.

### NOTES OF TWO CASES OF EXTRA-UTERINE PREGNANCY.

BY JAMES E. THOMPSON, M.B., B.S., LOND.; F.R.C.S., ENG.
Professor of Surgery, University of Texas.

[Read before Section on Gynecology, Texas State Medical Association.]

I HAVE reported the following two cases of extra-uterine pregnancy partly to place them on record, but mainly to excite discussion and bring forth the experiences of the members of this section on this interesting subject.

Being engaged, as I am, in general surgical practice, these cases come to me in consultation only, and I have not the chance that a general practitioner has of watching their development through the various phases. It is not my intention to enter exhaustively into the subject of tubal gestation, but to proceed directly to a description of the two cases I have operated on during the past two years.

CASE I.—Mrs. F., age 24, multipara. Occurred in the practice of Dr. Randall, of this city.

The menstrual period which was due about the middle of December, 1892, was missed, but in January, 1893, she passed a complete decidual cast of the uterine cavity (at least her description of it so tallied). Since then the menstrual flow has been seen repeatedly, at irregular intervals. She suffered intensely from an agonizing pain in the left groin, which confined her to bed; and, since that time, pains less intense than the first have repeatedly prostrated her. In the last two months, the tumor, which was examined by Dr. Randall, has doubled in size.

*Examination* revealed a swelling on the left side of the uterus, almost filling the pelvis. It was about the size of an orange, could be easily palpated between the two hands, and was distinctly fluctuant, elastic and tender. The uterus, which was pushed to the right, was freely movable and free from swelling, while a sound passed into the uterine cavity gave a length of three and a half inches. In addition to these points, the breasts were somewhat enlarged, and numerous pigmented spots were present on the forehead.

*Operation* was conducted under chloroform, and a median suprapubic incision was employed. The tumor was connected with the outer end of the left fallopian tube. A few adhesions, which existed behind the swelling, were soon broken down, the uterine end of the fallopian tube severed between ligatures, and the swelling lifted from it bed towards the ovarian attachments, which were likewise divided, and the mass shelled out. There was a little troublesome bleeding from the side of the uterus, but this was soon checked. A drainage tube was inserted into Douglas' pouch.

The after treatment was straightforward and uneventful. Temperature remained normal throughout. Pulse reached 102 during the first night, but afterwards remained at 80. Throughout the case, there was no pain, no vomiting, and nothing to excite apprehension.

The specimen removed, on examination, proved to be an intact gestation sac, occurring in an ovarian* pouch, as the diagram will show, a condition that has been suspected but not previously described. No fœtus was found, and no mole, while microscopic specimens of the sac wall failed to show evidence of placental villi.

CASE II.—Mrs. S., multipara, age 23. Had been married for two years, but had not previously been pregnant. The menstrual period which was due on October 25, 1894, was missed, but came on three days later, and was very profuse. The next period came on November 28, and during the interval an abundant discharge of watery, black fluid, with fœtid odor, had passed from the vagina. On November 15, she was examined at

---

*Bland Sutton. Diseases of Ovaries and Fallopian tubes, pp. 111 and 332.

CASE I.—PREGNANCY IN OVARIAN POUCH.

Hoboken, and her case pronounced to be one of extra-uterine pregnancy; and soon after the examination she suffered from intense pain in the right side, which has continued ever since, with remissions. She has never passed a recognizable decidua.

*Examination* revealed a swelling on the right side of the uterus, freely movable, circumscribed, and excessively tender.

It had no intimate connection with the uterus, which was appreciably elongated.

The temperature hovered about 100° F. There was no enlargement of the breasts, and no pigment spots. The diagnosis was a tentative one, and lay between pyo-salpinx and extra-uterine pregnancy.

*Operation* (Dr. Randall assisting) under chloroform, a median supra-pubic incision being employed.

As soon as the abdominal cavity was opened, the pelvis was found full of extravasated blood, mostly fluid, but containing numerous flocculated clots. These were cleared out, and a rent discovered on the posterior surface of the right fallopian tube, at its outer end. The fallopian tube, which was greatly enlarged, was removed in toto, and the abdominal cavity cleaned out and completely closed.

The patient suffered from intense nausea for three days, but eventually made a very quick convalescence, leaving hospital in three weeks.

*Examination* of the specimen showed a tube dilated with clotted blood, with a small rent near the fimbriated extremity, through which the blood which filled the pelvis had flowed. Examination of the blood showed a fleshy mole about the size of a small almond, which had evidently escaped into the abdominal cavity at the time of rupture, probably soon after she was examined at Hoboken.

Microscopic examination of the mole, and of the fallopian tube, failed to reveal any evidences of placental villi.

It will be seen from these cases that, although our evidence of extra-uterine pregnancy may be complete, both clinically and pathologically, from a gross examination, it may fail signally when we come to a minute microscopical examination. This may be due to one of two causes: (1) either the microscopic sections have failed to go through the chorionic villi, or (2) the villi may have become disintegrated. The chorionic villi are usually recognizable under the low power, and on section show an external layer of epithelial like cells, the central space being

CASE II.—RUPTURED TUBAL PREGNANCY.

occupied by irregular shaped cells. Under a high power the cells of the external layer are perfectly cubical.*

The tubal mole, or apoplectic ovum, found in case 2, was exactly similar to that figured by Bland Sutton, p. 319. Also, according to Cullingworth, the majority of cases of extra-uterine pregnancy, where rupture has occurred in the early months, will fail to reward a search for placental villi, and therefore absence of villi is no proof that the hæmato-sapinx has any other cause than extra-uterine pregnancy.

*Bland Sutton. Diseases of Ovaries and Fallopian Tubes, p. 320.

## Figures and Tables

**CASE I. f1:**
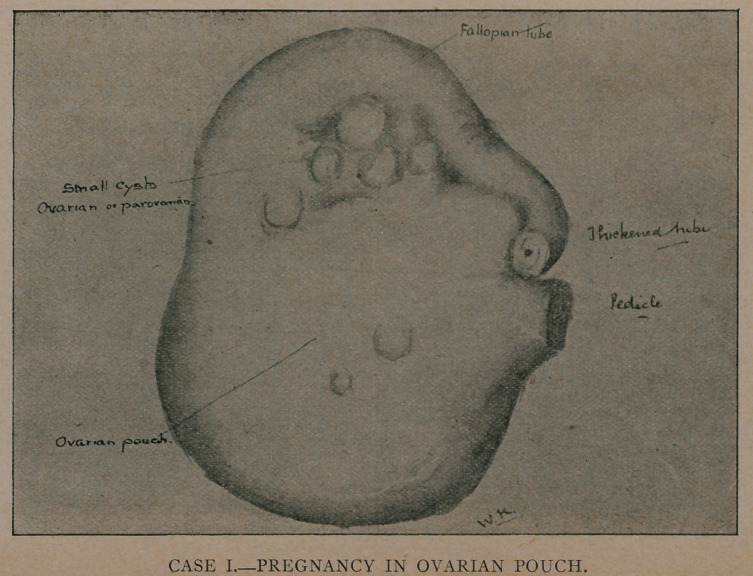


**CASE II. f2:**